# The impact of parent–child interaction and adolescent deviant behavior: the sequential mediation of depression and deviant peer affiliation

**DOI:** 10.3389/fpsyg.2025.1713762

**Published:** 2026-01-05

**Authors:** Yijie Wang, Juan Xu, Min Feng, Zhongdong Zhai, Ning Zou

**Affiliations:** 1 School of Education, Ludong University, Yantai, China; 2 Institute for Education and Treatment of Problematic Youth, Ludong University, Yantai, China; 3 The National Police University for Criminal Justice, Central Judicial Police College, Baoding, China; 4 Office of Development and Planning, Ludong University, Yantai, China

**Keywords:** adolescent deviant behavior, depression, deviant peer affiliation, parent–child interaction, sequential mediation

## Abstract

**Background:**

Adolescent deviant behavior in China—including aggression, truancy, and substance use—poses substantial risks for mental health, violations of social norms, and later criminal involvement. Prior studies have examined parent–child relationships, depressive symptoms, and deviant peer affiliation, but these factors are often treated in isolation. Evidence based on nationally representative data is still limited regarding how everyday parent–child interaction is linked to deviant behavior through both emotional states and peer networks within a single integrated model.

**Methods:**

Using Wave 2 (2014–2015) data from the China Education Panel Survey, we analyzed 8,294 junior high school students. Validated self-report scales were used to assess parent–child interaction, depressive symptoms, deviant peer affiliation, and deviant behavior. Correlation analyses and sequential mediation analyses (PROCESS Model 6) were conducted, controlling for gender, household registration type, and only-child status.

**Results:**

Parent–child interaction was negatively associated with depressive symptoms, deviant peer affiliation, and deviant behavior, whereas depressive symptoms and deviant peer affiliation were positively associated with deviant behavior. Mediation analyses indicated that parent–child interaction was indirectly related to deviant behavior via depressive symptoms, via deviant peer affiliation, and via the sequential combination of these two mediators. The final model explained 30.4% of the variance in deviant behavior.

**Conclusion:**

More frequent parent–child interaction is associated with lower levels of adolescent deviant behavior, in part because it is related to fewer depressive symptoms and less deviant peer affiliation. These findings support family-centered and mental health–informed strategies for preventing youth deviance and reducing later offending risk.

## 1 Introduction

Adolescent deviant behavior refers to actions that violate social norms or legal rules but do not yet meet the threshold for criminal conviction (Dullas et al., 2021; Sanches et al., 2016). As a critical risk indicator in social development, such behavior exerts long-term adverse effects on physical and mental health, academic performance, and social adjustment, and significantly predicts criminal involvement and maladaptive outcomes in adulthood (García-Fernández et al., 2022; Romm et al., 2021; Vierhaus et al., 2018). In recent years, deviant behavior among Chinese adolescents has become both more prevalent and more complex. Behavioral patterns have expanded beyond traditional school-based misconduct (e.g., truancy, fighting) to include cyberbullying, group conflicts, property offenses, and violent attacks with greater social harm (Dou et al., 2024; Guo, 2021).

Supreme People’s Procuratorate of the People’s Republic of China, (2025), procuratorial organs nationwide accepted 101,526 juvenile criminal suspects for review and prosecution in 2024, an increase of 4.3% compared with the previous year. Theft alone accounted for 33% of these cases. Between 2021 and 2023, courts handled 73,178 juvenile-related cases, among which left-behind children, single-parent families, and stepfamilies accounted for 22.94, 6.95, and 2.79% of defendants, respectively (Supreme People’s Procuratorate of the People’s Republic of China, 2025). However, in light of the “iceberg” metaphor (Lin et al., 2024), prosecuted criminal cases represent only the visible tip of a much larger base of behavioral problems. A large-scale national survey of Chinese children and adolescents reported a 35.1% prevalence of deviant behavior, which functions as a precursor to crime (Cui et al., 2021). This suggests that each formally prosecuted juvenile offender is backed by a much larger group of youths who are already engaging in early-stage deviant behaviors such as skipping class, fighting, and rule-breaking. Rising crime rates thus signal the expansion of this underlying base of deviance, making it imperative to examine how family supervision and developmental contexts shape adolescent deviant behavior as a priority for social governance and public policy.

This trend is closely linked to profound structural transformations in contemporary Chinese society. Rapid urbanization and large-scale population mobility have produced substantial numbers of left-behind and migrant children. Changes in marriage and family structures have led to increasing proportions of single-parent and stepfamilies. Intensified academic competition and work demands further compress the time and emotional resources available within families, rendering parent–child interaction more fragmented and instrumental (Cui et al., 2021; Shen et al., 2021). Under these conditions, the family’s role as an emotional safe haven and a core site of socialization is weakened, creating latent risks for the onset and accumulation of deviant behavior.

The Law on the Prevention of Juvenile Delinquency of the People’s Republic of China (revised in 2021) explicitly differentiates between “misconduct” (deviant behavior) and “serious misconduct” (acts violating the criminal law but below the age of criminal responsibility), and calls for graded and targeted interventions. Early identification and upstream intervention have become policy priorities (Yao, 2019). Within this policy context, clarifying how the family core system—especially parent–child interaction—shapes the development of adolescent deviant behavior has important theoretical and practical significance.

Although previous studies have extensively examined the effects of parenting styles and parent–child relationships on behavioral outcomes, much of this literature focuses on single pathways or treats parent–child interaction as a background variable rather than as an independent process. Few studies have systematically examined, within an integrated model, how concrete, everyday parent–child interaction influences deviant behavior through a continuous psychosocial mechanism that links emotional states and peer relationships. Drawing on nationally representative data, the present study constructs a sequential mediation model to explore the mechanisms underlying adolescent deviant behavior in the Chinese context from a combined perspective of social development and family systems, and to provide precise empirical evidence for policy implementation.

### 1.1 Parent–child interaction and deviant behaviors

Parent–child interaction refers to ongoing, bidirectional exchanges between parents and children in daily life—through conversation, shared activities, and emotional expression—and represents a key family environmental factor for adolescent social adjustment and behavioral development (Schneider et al., 2022; Zhou et al., 2020). Attachment theory posits that long-term, stable, and responsive parent–child interaction fosters secure attachment, which in turn supports emotional regulation and the internalization of social norms in adolescence (Kashyap et al., 2024). In contrast, conflictual, controlling, or neglectful interaction patterns can undermine emotional bonds and increase the risk of deviant and antisocial behavior (Walters, 2019; Yu, 2019).

Social control theory further emphasizes that frequent parent–child interaction strengthens emotional attachment to parents and encourages adolescents to voluntarily adhere to parental expectations and social norms, thereby reducing deviant behavior (Jianhua et al., 2025; Svensson et al., 2025). Empirical studies have generally found positive associations between supportive parent–child relationships and adolescent development (van Dijk et al., 2025). However, many studies operationalize the parent–child relationship using global indicators such as overall relationship quality or parenting style, or treat parent–child interaction as a background covariate rather than a distinct mechanism (Cadman et al., 2022; Zhu et al., 2022).

In the context of increasingly diverse family structures in China (e.g., left-behind, single-parent, and stepfamilies) and weakening family functioning, this gap in the literature is particularly salient. Judicial practice also indicates that juvenile offenders often report disrupted parent–child interaction and a lack of emotional support at home, highlighting the central role of parent–child interaction in the development of deviant behavior (Supreme People’s Procuratorate of the People’s Republic of China, 2025). Accordingly, we propose the following hypothesis: H1: More frequent parent–child interaction is associated with lower levels of adolescent deviant behavior.

### 1.2 The mediating role of depression

The influence of parent–child interaction on adolescent deviant behavior is not limited to behavioral supervision and norm enforcement; it may also operate indirectly through internal emotional processes, among which depression is a key psychological mechanism bridging family risk and deviant behavior (Ratliff et al., 2023).

From an attachment perspective, stable and responsive parent–child interaction provides adolescents with a sense of security and support in the family, fostering basic trust in self and others and reducing the risk of depression (Jianhua et al., 2025). Conversely, infrequent interaction, emotional coldness, or punitive parenting may undermine this sense of security, increase feelings of isolation and helplessness, and heighten vulnerability to depressive symptoms (Zhang et al., 2024).

Emotional security theory further suggests that in high-conflict or tense family environments, adolescents must continuously invest psychological resources in self-protection. The sustained emotional strain consumes regulatory resources and impairs their capacity for emotional regulation, thereby increasing the likelihood of depression (Yang et al., 2022).

**Figure 1:**
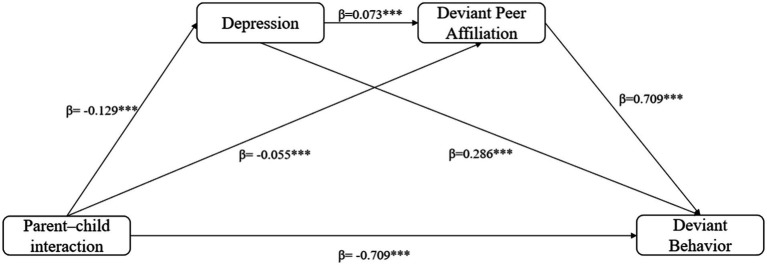
Path diagram for the final mediation model. ***p < 0.001.

Empirically, depression has been found to be positively associated with various forms of deviant behavior, including aggression, rule-breaking, and substance use. Adolescents experiencing depression often struggle with emotion regulation and behavioral inhibition and may resort to impulsive, oppositional, or high-risk behaviors as a way to temporarily alleviate negative affect, thereby increasing the risk of deviant behavior (Zheng et al., 2025). From a developmental psychopathology perspective, internalizing problems such as depression and externalizing problems such as deviant behavior frequently co-occur, and depression can precede and contribute to deviant behavior. Thus, we hypothesize: H2: Depression mediates the association between parent–child interaction and adolescent deviant behavior (Figure 1).

### 1.3 The mediating role of deviant peer affiliation

Beyond the family, peers constitute a crucial socialization context for the development of adolescent deviant behavior. Parent–child interaction may not only directly influence behavior but may also operate indirectly through peer relationships (Guo, 2022; de Maat et al., 2021).

Deviant peer affiliation refers to close association with peers who engage in antisocial or rule-breaking behaviors, and the resulting exposure to their values, cognitions, and behavioral patterns, which increases the risk of deviant behavior (Allen et al., 2021; Buil et al., 2017). Differential association theory posits that deviance is learned through interaction with deviant peers, as adolescents internalize their motivations and attitudes (Defoe et al., 2018).

From a social control perspective, weak emotional bonds within the family reduce informal social control and make adolescents more likely to seek emotional support and identity from peer groups, thereby increasing their risk of deviant involvement (Graaff et al., 2012). Empirical work has shown that deviant peer affiliation partially mediates the relation between parent–child interaction and deviant behavior (Branje, 2024), suggesting that family processes may shape the structure and quality of adolescents’ peer networks. Based on this literature, we propose: H3: Deviant peer affiliation mediates the association between parent–child interaction and adolescent deviant behavior.

### 1.4 The sequential mediation of depression and deviant peer affiliation

The detrimental effects of depression on adolescents’ peer relationships are well documented (Rodriguez et al., 2019). As noted, insufficient or low-quality parent–child interaction may foster feelings of emotional insecurity and thereby increase depressive symptoms (Kashyap et al., 2024; Yang et al., 2022). Adolescents with depression tend to withdraw from positive peer relationships and may instead gravitate toward deviant peer groups that appear to offer emotional support, even though these peers are characterized by problematic behaviors (Lindblom et al., 2018).

Nonformal social control perspectives suggest that when the family fails to provide adequate emotional bonds, adolescents become more susceptible to peer influences and more likely to deviate from social norms (Havewala et al., 2021). Empirical studies have shown that adolescents with depressive tendencies are more likely to associate with peers displaying antisocial behavior, which not only fails to alleviate negative emotions but may further exacerbate deviance (Archer et al., 2022).

In this way, depression and deviant peer affiliation may form a sequential mediating pathway linking parent–child interaction to deviant behavior. Specifically, insufficient emotional support in the family may foster depressive symptoms, which in turn increase the likelihood of affiliating with deviant peers; exposure to deviant peer norms then elevates the risk of deviant behavior. Thus, we hypothesize: H4: Depression and deviant peer affiliation sequentially mediate the association between parent–child interaction and adolescent deviant behavior.

### 1.5 The present study

In sum, although prior research has documented associations between parent–child interaction and adolescent social and behavioral development, the underlying mechanisms—particularly the roles of emotional processes and peer relationships—require more systematic examination. To address this gap, the present study uses nationally representative data from the China Education Panel Survey (CEPS) to construct a sequential mediation model. We investigate how parent–child interaction relates to adolescent deviant behavior and, in particular, how depression and deviant peer affiliation mediate this association.

By integrating family interaction, psychological adjustment, and peer relationships within a single model, this study seeks to refine the social–psychological mechanisms underlying adolescent deviant behavior in contemporary China and to provide more targeted theoretical and practical implications for family-based intervention and early prevention.

## 2 Materials and methods

### 2.1 Participants and procedures

Data for this study were drawn from the China Education Panel Survey (CEPS), organized and implemented by the China Survey and Data Center at Renmin University of China. CEPS is the first large-scale, nationally representative longitudinal survey targeting junior high school students in China (Zhong et al., 2022).

The baseline survey of CEPS (2013–2014 academic year) adopted a multi-stage stratified probability sampling strategy combined with probability proportional to size (PPS) sampling. Twenty-eight county-level units across the country were selected as primary sampling units. A total of 112 schools and 438 classes were subsequently sampled, yielding a baseline sample of approximately 20,000 students. This sample size was composed of two grade cohorts: Grade 7 and Grade 9. The survey collected rich and multidimensional information on students, including sociodemographic characteristics, hukou and migration status, developmental experiences (e.g., parental divorce or death), physical and mental health, parent–child interaction, school learning, extracurricular activities, relationships with teachers and classmates, and social behavior. These data provide comprehensive support for the present study.

In the 2014–2015 academic year, only Grade 7 students from the baseline survey (who had progressed to Grade 8 at follow-up) were tracked. Grade 9 students at baseline were not included in subsequent follow-ups because they had moved on to high school or vocational school, entered the labor market, or migrated across regions. As a result, 9,449 students were successfully followed up. The reduction in sample size thus stemmed primarily from the survey design and changes in tracking eligibility, rather than from large-scale attrition within the sample. By Wave 2, all students had advanced from Grade 7 to Grade 8. Although CEPS is a longitudinal survey, the present study employs cross-sectional analyses based on Wave 2 data for two reasons. First, Grade 8 represents a critical developmental period in which deviant behavior tends to peak. Drawing on our research team’s long-term fieldwork in juvenile correctional facilities, women’s prisons, and special schools across several provinces (e.g., Shandong, Zhejiang, Guizhou, Fujian), as well as prior studies, Grade 8 is often a pivotal stage in the escalation of adolescent deviant behavior (Yan and Zhang, 2025). Wave 2 data therefore better capture the behavioral risks of interest. Second, the Wave 2 questionnaire features more refined and complete measurement tools than the baseline, offering improved assessment of key constructs.

Regarding missing data, given the large overall sample size and the low proportion of missingness on core variables, listwise deletion was primarily used to preserve the authenticity of observed responses. Additionally, we conducted robustness checks using multiple imputation. The results showed that the main path coefficients and significance levels were highly consistent across the two approaches, indicating that missing data did not materially bias the findings (refer to the Supplementary material). The final analytic sample consisted of 8,294 students who had complete data on all key study variables.

### 2.2 Measures

#### 2.2.1 Parent–child interaction

Parent–child interaction was assessed with a 16-item scale (item codes: w2a2001–w2a2006, w2a2101a–w2a2104b, w2a22–w2a23) that captures the frequency of interaction between adolescents and their parents. Items cover academic management (e.g., homework, examinations), everyday behavioral regulation (e.g., friendships, Internet use, clothing), and parent–child communication (e.g., discussing school life, interpersonal relationships, and personal concerns). Responses are rated on a 3-point Likert scale (1 = never, 3 = often). Item scores were summed to create a total score, with higher scores indicating more frequent parent–child interaction. This measure has been widely used and validated in CEPS-based studies (Dou et al., 2025). Internal consistency in the present sample was good (Cronbach’s *α* = 0.858).

#### 2.2.2 Depression

Depressive symptoms during the past 7 days were measured using a five-item scale (item codes: w2c2501, w2c2503–w2c2506), covering feelings of depression, unhappiness, lack of interest in life, lack of motivation, and sadness. Responses are rated on a 5-point Likert scale (1 = never, 5 = always). Item scores were summed, with higher scores indicating more severe depressive symptoms. This measure has demonstrated good reliability and validity in CEPS-based research (Wang and Sun, 2024). In the present study, internal consistency was excellent (Cronbach’s *α* = 0.901).

#### 2.2.3 Deviant peer affiliation

Deviant peer affiliation was assessed using a six-item scale (item codes: w2d1104–w2d1110). Respondents reported whether their close friends engaged in behaviors such as truancy, school rule violations leading to disciplinary sanctions, fighting, drinking, frequent visits to Internet cafés/game halls, and early romantic relationships or dropping out of school. Responses are rated on a 3-point Likert scale (1 = none, 3 = many). Item scores were summed, with higher scores indicating greater exposure to deviant peers. This measure has been widely used and shown good psychometric properties in CEPS-based research (Lu, 2024). Internal consistency in the present sample was good (Cronbach’s *α* = 0.870).

#### 2.2.4 Deviant behavior

Adolescent deviant behavior over the past year was measured using a 10-item scale (item codes: w2d0201–w2d0210). Items assess behaviors such as swearing, quarreling, fighting, bullying classmates, irritability, inattention, truancy, cheating, smoking/drinking, and excessive time spent in Internet cafés. Responses are rated on a 5-point Likert scale (1 = never, 5 = frequently). Item scores were summed, with higher scores indicating greater involvement in deviant behavior. This scale has been widely validated in CEPS-based research (Li, 2022; Cai, 2021). Internal consistency in the present sample was good (Cronbach’s α = 0.818).

### 2.3 Statistical analyses

Data were screened and coded using SPSS 26.0. To assess potential common method bias, we conducted a principal component analysis (Harman’s single-factor test). Descriptive statistics (means and standard deviations) were computed for all demographic and study variables. Partial correlation analyses were performed to examine associations among parent–child interaction, depressive symptoms, deviant peer affiliation, and deviant behavior while controlling for gender, household registration type, and only-child status.

Sequential mediation analyses were conducted in SPSS 26.0 using the PROCESS macro (Model 6) (Haslam and Taylor, 2022). A bias-corrected bootstrapping procedure with 5,000 resamples was used to estimate indirect effects. Indirect effects were considered statistically significant if the 95% confidence interval did not include zero (Table 1).

**Table 1:** Demographic variables of participants and their distribution proportions.

Category	Subcategory	Count(N)	Percentage (%)
Registered residence location	Within county/district	6,812	82.1
	Outside county/district	1,482	17.9
Hukou type	Agricultural	4,404	53.1
	Non-agricultural	2,232	26.9
	Residential	1,642	19.8
	No Hukou	16	0.2
Only child	Yes	3,659	44.1
	No	4,635	55.9
Parents currently married	Yes	613	7.4
	No	7,681	92.6
Parents living together	YES	7,223	87.1
	No	1,071	12.9
Parents divorced	Yes	536	6.5
	No	7,758	93.5
Custodial parent after divorce	Father	279	3.4
	Mother	230	2.8
	Other	11	0.1
Father deceased	Yes	8,175	98.6
	No	119	1.4
Mother deceased	Yes	75	0.9
	No	8,219	99.1

## 3 Results

### 3.1 Common method bias

Harman’s single-factor test was used to assess common method bias. The first unrotated factor accounted for 32.67% of the total variance, below the commonly used 40% threshold, suggesting that common method bias was unlikely to substantially influence the results (Luo et al., 2023).

### 3.2 Demographic characteristics

The final sample included 8,294 students. Most held local household registration within their county or district (82.1%, *n* = 6,812), while 17.9% (*n* = 1,482) were registered elsewhere. Regarding hukou type, 53.1% (*n* = 4,404) had agricultural hukou, 26.9% (*n* = 2,232) non-agricultural, 19.8% (*n* = 1,642) residential, and 0.2% (*n* = 16) had no hukou.

In terms of family structure, 44.1% (*n* = 3,659) were only children. Most participants reported that their parents were currently married (92.6%, *n* = 7,681) and cohabiting (87.1%, *n* = 7,222), whereas 6.5% (*n* = 536) reported parental divorce. Among those with divorced parents, 3.4% (*n* = 279) lived with their father, 2.8% (*n* = 230) with their mother, and 0.1% (*n* = 11) with other guardians. A small proportion reported a deceased father (1.4%, *n* = 119) or deceased mother (0.9%, *n* = 75). Overall, the sample was characterized by predominantly intact family structures and local household registration.

### 3.3 Correlation analysis

Table 2 presents partial correlations among the main study variables, controlling for gender, household registration type, and only-child status. Parent–child interaction was significantly and negatively correlated with depression (*r* = −0.166, *p* < 0.001), deviant peer affiliation (*r* = −0.171, *p* < 0.001), and deviant behavior (*r* = −0.251, *p* < 0.001), indicating that more frequent parent–child interaction is associated with lower levels of depression, deviant peer affiliation, and deviant behavior. Depression was positively correlated with deviant peer affiliation (*r* = 0.175, *p* < 0.001) and deviant behavior (*r* = 0.361, *p* < 0.001). Deviant peer affiliation was also positively correlated with deviant behavior (*r* = 0.408, *p* < 0.001), suggesting that adolescents with greater exposure to deviant peers are more likely to engage in deviant behavior.

**Table 2:** Means, standard deviations, and partial correlations among study variables.

Variable	*M*	SD	1	2	3	4
1. Parent–child interaction	35.48	5.87	1			
2. Depression	10.77	4.52	−0.166***	1		
3. Deviant peer affiliation	8.20	2.24	−0.171***	0.175***	1	
4. Deviant behavior	15.57	4.73	−0.251***	0.361***	0.408***	1

Control variables: gender, household registration type, and only-child status. *N*, 8,294; *M*, mean; SD, standard deviations. *p < 0.05, **p < 0.01, ***p < 0.001.

### 3.4 Mediation analysis

Table 3 summarizes the mediation analysis results. Parent–child interaction was significantly and negatively associated with depression (*β* = −0.129, *p* < 0.001), deviant peer affiliation (*β* = −0.055, *p* < 0.001), and deviant behavior (*β* = −0.118, *p* < 0.001). Depression was positively associated with deviant peer affiliation (*β* = 0.073, *p* < 0.001) and deviant behavior (*β* = 0.286, *p* < 0.001), while deviant peer affiliation was strongly associated with deviant behavior (*β* = 0.709, *p* < 0.001). The model explained 30.4% of the variance in deviant behavior.

**Table 3:** Mediation analysis results.

	Depression β(t)	Deviant peer affiliation β(t)	Deviant behavior β(t)	Total effect on deviant behavior β(t)
Constant	14.079*** (35.10)	10.523*** (51.29)	11.537*** (26.27)	–
Parent–child interaction	−0.129*** (−15.32)	−0.055*** (−13.42)	−0.118*** (−15.46)	−0.118*** (−15.46)
Depression		0.073*** (13.92)	0.286*** (28.95)	
Deviant peer affiliation			0.709*** (34.71)	
*R* ^2^	0.034	0.104	0.304	0.304
*F*	72.67***	192.74***	602.21***	602.21***

Control variables: gender, household registration type, and only-child status. *N* = 8,294. *p < 0.05, **p < 0.01, ***p < 0.001.

Table 4 presents the specific mediation pathways. The indirect effect of parent–child interaction on deviant behavior via depression was significant (*β* = −0.037, 95% CI [−0.043, −0.031]), as was the indirect pathway via deviant peer affiliation (*β* = −0.039, 95% CI [−0.048, −0.031]). The sequential mediation pathway (parent–child interaction → depression → deviant peer affiliation → deviant behavior) was also significant (*β* = −0.007, 95% CI [−0.009, −0.005]). The total indirect effect was −0.082 (95% CI [−0.093, −0.072]), and the total effect of parent–child interaction on deviant behavior was −0.200 (95% CI [−0.211, −0.189]), indicating that both individual and sequential mediation pathways contribute meaningfully to the overall association between parent–child interaction and adolescent deviant behavior.

**Table 4:** Path analysis results.

Effect type	Path	Estimate	SE	*t*	*p*	95% CI lower	95% CI upper
Direct effect	Parent–child interaction → depression	−0.129	0.008	−15.32	0	−0.145	−0.112
	Depression → deviant peer affiliation	0.073	0.005	13.92	0	0.063	0.083
	Parent–child interaction → deviant peer affiliation	−0.055	0.005	−13.42	0	−0.062	−0.046
	Depression → deviant behavior	0.286	0.010	28.95	0	0.267	0.306
	Deviant peer affiliation → deviant behavior	0.709	0.042	34.79	0	0.672	0.752
	Parent–child interaction → deviant behavior	−0.118	0.008	−15.46	0	−0.133	−0.103
Indirect effect	Parent–child interaction → depression → deviant behavior	−0.037	0.003	–	–	−0.043	−0.031
	Parent–child interaction → deviant peer affiliation → deviant behavior	−0.039	0.004	–	–	−0.048	−0.031
	Parent–child interaction → depression → deviant peer affiliation → deviant behavior	−0.007	0.001	–	–	−0.009	−0.005
Total indirect	Parent–child interaction → deviant behavior (total indirect effect)	−0.082	0.006	–	–	−0.093	−0.072
Total effect	Parent–child interaction → deviant behavior (total effect)	−0.200	0.011	–	–	−0.211	−0.189

Control variables: gender, household registration type, and only-child status. *N* = 8,294. *p < 0.05, **p < 0.01, ***p < 0.001.

## 4 Discussion

Using nationally representative data from CEPS, this study confirmed a significant negative association between parent–child interaction frequency and adolescent deviant behavior and demonstrated that depression and deviant peer affiliation jointly form a sequential mediating pathway. Unlike prior research that typically focused on a single mechanism, our integrated model illustrates how deviant behavior emerges as a continuous process that begins with deficits in family interaction, progresses through internal emotional distress, and ultimately manifests as maladaptive socialization with deviant peers. These findings not only corroborate classical developmental theories but also provide a context-sensitive understanding of behavioral risk among Chinese adolescents in a period of rapid social transformation.

### 4.1 Parent–child interaction and deviant behavior

Our results showed that parent–child interaction significantly and negatively predicted adolescent deviant behavior, consistent with domestic and international evidence on the protective role of supportive parent–child relationships against externalizing problems (Branje, 2024; Lavner et al., 2021). This finding aligns with Hirschi’s social control theory, which posits that stronger emotional bonds with parents increase youths’ attachment to conventional norms and raise the psychological costs of rule-breaking (Niu et al., 2023).

Compared with studies that focus on relatively static indicators such as global relationship quality or parenting style, the present study used a comprehensive measure of parent–child interaction frequency that captures academic management, behavioral regulation, and emotional communication. This approach better reflects the intensity of everyday interactions within the family. In the context of rapid urbanization, accelerated work rhythms, and widespread time scarcity among parents in China, everyday parent–child interaction has become a scarce resource (Gu, 2021). Our findings suggest that even under conditions of compressed shared time and fragmented interaction opportunities, sustained parental involvement in academic, daily, and emotional domains exerts a robust buffering effect, partially offsetting the adverse impact of structural changes on adolescent behavioral development. This provides large-sample evidence for conceptualizing parent–child interaction as a key “family buffer” in times of social transition.

### 4.2 The mediating role of depression

We found that depression significantly mediated the association between parent–child interaction and adolescent deviant behavior: lower levels of parent–child interaction were associated with higher levels of depressive symptoms, which in turn predicted greater deviant behavior (Yu et al., 2025). This indicates that parent–child interaction not only has a direct negative association with deviant behavior but also exerts an indirect effect via depressive symptoms, forming a key emotional pathway linking family processes to behavioral problems (Defoe et al., 2023). These results are consistent with the developmental hypothesis that family dysfunction fosters internalizing problems, which subsequently increase the risk of externalizing co-occurrence.

Theoretically, this mediation effect can be understood through both attachment theory and emotional security theory. Frequent, responsive interaction helps create a stable and predictable family climate in which adolescents can readily access emotional support and clarification when facing academic or interpersonal stress, thereby reducing the accumulation of negative affect and the risk of depression (Spruit et al., 2020). Conversely, when interaction is infrequent or instrumental and focused largely on performance, adolescents’ emotional needs may be overlooked, heightening vulnerability to depression. Existing research has also shown that adolescents with depression exhibit greater affective instability and weaker behavioral inhibition, and are more likely to use truancy, rule-breaking, or high-risk activities to temporarily relieve emotional burden, thereby increasing the likelihood of deviant behavior (Sims et al., 2024).

In China’s highly competitive educational environment, this mechanism is particularly salient. Parent–child interaction is at risk of becoming “instrumentalized” around academic achievement, with emotional communication sidelined (Quach et al., 2015). Our findings highlight depression as a critical mediator between parent–child interaction and deviant behavior, suggesting that interventions should move beyond a narrow focus on discipline to address adolescents’ emotional needs and mental health.

### 4.3 The mediating role of deviant peer affiliation

Deviant peer affiliation was also found to significantly mediate the association between parent–child interaction and deviant behavior. This finding strongly supports Sutherland’s differential association theory, which conceptualizes deviant behavior as a learned outcome of interaction with deviant peers who provide the primary environment for exposure to deviant definitions and practices. It is also consistent with empirical research showing that when “push factors” within the family intensify, adolescents are more likely to seek alternative social bonds outside the family (Guo, 2022; Yang et al., 2022).

By jointly examining family processes and peer contexts, this study demonstrates that parent–child interaction shapes adolescents’ peer choices. When interaction is frequent and supportive, the family can provide a sufficient sense of belonging and normative guidance, promoting affiliation with prosocial peers and reducing exposure to deviant peer networks. In contrast, when parent–child interaction is lacking, adolescents may be driven to seek acceptance and recognition from deviant peer groups. In the current era of digitalization and widespread social media, barriers to contact with deviant peer groups have been greatly lowered, including via online spaces. This amplifies the urgency of the family’s role as the first line of defense against deviant peer influences (Cioban et al., 2021; McCuddy et al., 2024).

### 4.4 The sequential mediation of depression and deviant peer affiliation

Our study further elucidated a sequential pathway linking parent–child interaction, depression, deviant peer affiliation, and deviant behavior. Beyond demonstrating associations among these variables, the model depicts a developmental sequence that begins with insufficient emotional support in the family, progresses through internal emotional dysregulation, and culminates in deviant peer affiliation and behavioral deviation. This extends prior research by revealing a more fine-grained process of risk transmission (Oliveira et al., 2024; Rodriguez et al., 2019).

Specifically, when parent–child interaction is infrequent or lacking in responsiveness, adolescents may lack effective “emotional relief valves” in the face of academic and developmental pressures, leading to the accumulation of negative affect and elevated depressive symptoms (Yu et al., 2024). Importantly, this internal distress does not remain confined to the psychological domain; rather, it covaries with changes in social trajectories. Theories of peer selection suggest that adolescents with depression often struggle with low self-worth and impaired social skills, are marginalized in mainstream peer groups, and are more likely to be drawn into deviant peer networks (Chen et al., 2023). Within such groups, initially seeking emotional comfort may become tightly intertwined with exposure to deviant subcultural norms. Through observational learning and peer reinforcement, deviant behaviors can gradually shift from serving as outlets for emotional expression to functioning as instruments for gaining group recognition (Trucco et al., 2011).

These findings suggest that deviant behavior is not the product of any single factor but rather the outcome of complex interactions among family systems, emotional processes, and peer systems. In the contemporary Chinese context, particularly for left-behind children and adolescents in high-risk environments, this sequential pathway underscores the importance of early identification and intervention targeting internalizing distress. Rather than focusing solely on behavioral control at the endpoint, prevention efforts should aim to interrupt the trajectory at earlier stages—by addressing depressive symptoms and enhancing emotional support—to reduce the likelihood that adolescents turn to deviant peers and progress into more serious deviant behavior.

## 5 Limitations

Despite its contributions, this study has several limitations. First, the use of cross-sectional data precludes precise conclusions about temporal dynamics and causal relationships among the variables (VanderWeele, 2015). Future research should employ longitudinal and experimental designs to test the temporal ordering and causal mechanisms underlying these associations.

Second, the sample consists of Chinese adolescents. Although the findings have strong representativeness for this population, further studies are needed in diverse cultural and social contexts to establish the generalizability of the model.

Finally, although we focused on depression and deviant peer affiliation as mediators, other factors—such as school climate, family socioeconomic status, and community support—may also play important roles in the development of deviant behavior. Future research should broaden the model to incorporate these additional contextual and structural variables.

## 6 Implications

This study offers several concrete implications for policy and practice in the Chinese context. First, in light of the fragmentation and instrumentalization of parent–child interaction, family education programs should prioritize the quality rather than the mere quantity of interaction. Policymakers and community practitioners should promote the concept of high-quality parental involvement, encouraging parents to reduce narrow academic monitoring and increase emotional support and open communication within limited interaction time. For dual-earner and left-behind families in particular, simple and feasible strategies to enhance everyday parent–child interaction should be promoted to restore the family’s function as an emotional safe haven.

Second, given the “springboard” role of depression in the pathway to deviant behavior, schools and communities should build destigmatized mental health monitoring systems. Education authorities should integrate mental health screening into routine student health examinations, with a particular focus on adolescents who have not yet engaged in overt misconduct but show signs of low mood or social withdrawal. Evidence-based interventions such as cognitive–behavioral therapy (CBT) can help adolescents reconstruct negative cognitions and address emotional problems before they manifest as deviant behavior.

Third, to address peer risks in the digital era, schools and communities should provide attractive alternative sources of belonging. Simple prohibitions (e.g., banning Internet use or restricting friendships) often have limited effectiveness under current conditions. More promising strategies include providing structured, prosocial activities—such as sports, arts clubs, and volunteer service—to foster positive peer cultures and experiences of achievement. These can counterbalance the appeal of deviant peer groups and online deviant subcultures, thereby improving the quality of adolescents’ social environments.

Finally, for adolescents already involved with deviant peers, dual-track interventions are needed. In addition to behavior-focused interventions for adolescents themselves, it is essential to empower parents by helping them improve the quality of the parent–child relationship and gradually reclaim positive influence. Such systemic approaches may help break vicious cycles and support the resocialization of at-risk youth.

## 7 Conclusion

Drawing on a nationally representative sample, this study constructed and validated a sequential mediation model in which parent–child interaction influences adolescent deviant behavior through depression and deviant peer affiliation. The results show that parent–child interaction not only exerts a direct protective effect on deviant behavior but also indirectly reduces deviance by alleviating internalizing distress and limiting exposure to deviant peers.

These findings suggest that deviant behavior often originates in insufficient support within family interactions and unfolds through a stepwise process involving emotional problems and peer influences. In the context of rapid social change and evolving family structures in China, the study underscores the foundational role of parent–child interaction in preventing adolescent deviant behavior and provides empirical support for legal and policy frameworks that emphasize early intervention. Effective prevention efforts should simultaneously enhance the quality of family interaction, strengthen mental health support, and guide peer relationships in order to reduce the risk of deviant behavior at an early stage.

## Data Availability

Publicly available datasets were analyzed in this study. This data can be found at: http://ceps.ruc.edu.cn/.
