# Supplementary material for: The impact of parent–child interaction and adolescent deviant behavior: the sequential mediation of depression and deviant peer affiliation
Source: Front Psychol. 2026 Jan 5;16:1713762. doi: 10.3389/fpsyg.2025.1713762 (PMC12812652; doi:10.3389/fpsyg.2025.1713762)
Supplement: Supplementary file 1 [file Supplementary_file_1.docx]

# ****Supplementary Materials****

## ****Table S1 Direct Path Estimates and Standard Errors: Original vs. Pooled (MI)****

| Path | Original Coeff. | MI1 | MI2 | MI3 | MI4 | MI5 | Pooled Coeff. | Original SE | Pooled SE |
| --- | --- | --- | --- | --- | --- | --- | --- | --- | --- |
| Parent-child → Depression | -0.129 | -0.128 | -0.13 | -0.129 | -0.128 | -0.129 | -0.129 | 0.008 | 0.008 |
| Depression → Deviant Peers | 0.073 | 0.072 | 0.074 | 0.073 | 0.073 | 0.074 | 0.0732 | 0.005 | 0.005 |
| Parent-child → Deviant Peers | -0.055 | -0.054 | -0.056 | -0.055 | -0.055 | -0.054 | -0.055 | 0.005 | 0.005 |
| Depression → Deviant Behavior | 0.286 | 0.285 | 0.287 | 0.286 | 0.285 | 0.288 | 0.286 | 0.01 | 0.01 |
| Deviant Peers → Deviant Behavior | 0.709 | 0.708 | 0.71 | 0.709 | 0.707 | 0.711 | 0.709 | 0.042 | 0.043 |
| (Direct) Parent-child → Deviant Behavior | -0.118 | -0.117 | -0.119 | -0.118 | -0.117 | -0.118 | -0.118 | 0.008 | 0.008 |

Covariates: gender, household registration type, only-child status

## ****Table S2 Indirect and Total Effects: Original vs. Pooled (MI)****

| Indirect Path | Original Est. | MI1 | MI2 | MI3 | MI4 | MI5 | Pooled Est. | Original 95% CI | Pooled 95% CI |
| --- | --- | --- | --- | --- | --- | --- | --- | --- | --- |
| Ind1: Parent-child → Depression → Deviant Behavior | -0.037 | -0.036 | -0.038 | -0.037 | -0.037 | -0.037 | -0.037 | [-0.043, -0.031] | [-0.043, -0.031] |
| Ind2: Parent-child → Deviant Peers → Deviant Behavior | -0.039 | -0.038 | -0.04 | -0.039 | -0.039 | -0.039 | -0.039 | [-0.048, -0.031] | [-0.048, -0.030] |
| Ind3: Parent-child → Depression → Deviant Peers → Deviant Behavior | -0.007 | -0.007 | -0.007 | -0.007 | -0.007 | -0.007 | -0.007 | [-0.009, -0.005] | [-0.009, -0.005] |
| Total Indirect | -0.082 | -0.081 | -0.083 | -0.082 | -0.081 | -0.084 | -0.0822 | [-0.093, -0.072] | [-0.094, -0.071] |
| Total Effect | -0.2 | -0.199 | -0.201 | -0.2 | -0.198 | -0.202 | -0.2 | [-0.211, -0.189] | [-0.212, -0.188] |

Covariates: gender, household registration type, only-child status
